# Understanding Needs of People With Alzheimer’s Disease: The Role of Caregiver Perspective Taking

**DOI:** 10.1093/geroni/igaf122.288

**Published:** 2025-12-31

**Authors:** Meng Huo, Rose Tran, Emily Mroz

**Affiliations:** University of California, Davis, Davis, California, United States; University of California, Davis, Davis, California, United States; Emory University, Atlanta, Georgia, United States

## Abstract

Despite receiving intensive support from caregivers, people living with Alzheimer’s disease (PLWD) still face unmet care needs that exacerbate their own functional declines and increase caregiver burden. Unmet needs emerge when caregivers and PLWD are misaligned about PLWD’s needs. We conducted a mixed-methods study with 67 couples in which one person had mild-to-moderate Alzheimer’s disease. Both spouses completed semi-structured interviews to rate PLWD’s needs across 16 areas (e.g., self-care, memory, company) using the modified Camberwell Assessment of Need for the Elderly (CANE); they also discussed how they communicated about those needs. We examined caregivers’ perspective taking—their ability to understand PLWD’s thoughts and feelings and observed a negative association between perspective taking and spousal discrepancies in PLWD’s needs. We then conducted a thematic analysis of caregivers’ qualitative descriptions about communication strategies about PLWD’s needs. When caregivers had high perspective taking, spouses described engaging in open, effective communication that fostered a shared understanding of caregiving expectations and caregivers felt they adopted PLWD-centered strategies that sustained or enhanced PLWD’s sense of competence. When caregivers had low perspective taking, spouses described disagreeing on the care PLWD needed and some caregivers acknowledged being transparently frustrated in conversations with PLWD. Findings illustrate how and why caregivers’ perspective taking can direct their efforts to understand and meet the needs of PLWD: perspective taking can facilitate couple communication and enhance caregivers’ perceptions of and responses to PLWD’s needs. These insights can inform interventions to improve the quality of dementia care and promote health in caregiving dyads.

